# Ecosystem and soil respiration radiocarbon detects old carbon release as a fingerprint of warming and permafrost destabilization with climate change

**DOI:** 10.1098/rsta.2022.0201

**Published:** 2023-11-27

**Authors:** Edward A. G. Schuur, Caitlin Hicks Pries, Marguerite Mauritz, Elaine Pegoraro, Heidi Rodenhizer, Craig See, Chris Ebert

**Affiliations:** ^1^ Center for Ecosystem Science and Society, and Department of Biological Sciences, Northern Arizona University, Flagstaff, AZ 86011, USA; ^2^ Department of Biological Sciences, Dartmouth College, Hanover, NH 03755, USA; ^3^ Biological Sciences, University of Texas at El Paso, 500 West University Avenue, El Paso, TX 79902, USA; ^4^ Climate and Ecosystem Sciences Division, Lawrence Berkeley National Lab, Berkeley, CA, USA; ^5^ Woodwell Climate Research Center, Falmouth, MA 02540, USA

**Keywords:** arctic tundra, permafrost soil carbon, climate change, isotopes, radiocarbon, ecosystem respiration

## Abstract

The permafrost region has accumulated organic carbon in cold and waterlogged soils over thousands of years and now contains three times as much carbon as the atmosphere. Global warming is degrading permafrost with the potential to accelerate climate change as increased microbial decomposition releases soil carbon as greenhouse gases. A 19-year time series of soil and ecosystem respiration radiocarbon from Alaska provides long-term insight into changing permafrost soil carbon dynamics in a warmer world. Nine per cent of ecosystem respiration and 23% of soil respiration observations had radiocarbon values more than 50‰ lower than the atmospheric value. Furthermore, the overall trend of ecosystem and soil respiration radiocarbon values through time decreased more than atmospheric radiocarbon values did, indicating that old carbon degradation was enhanced. Boosted regression tree analyses showed that temperature and moisture environmental variables had the largest relative influence on lower radiocarbon values. This suggested that old carbon degradation was controlled by warming/permafrost thaw and soil drying together, as waterlogged soil conditions could protect soil carbon from microbial decomposition even when thawed. Overall, changing conditions increasingly favoured the release of old carbon, which is a definitive fingerprint of an accelerating feedback to climate change as a consequence of warming and permafrost destabilization.

This article is part of the Theo Murphy meeting issue ‘Radiocarbon in the Anthropocene’.

## 1. Introduction

Global surface temperatures are over 1°C warmer at present relative to the start of the industrial period due to greenhouse gas emissions and other human activities [1]. In northern high latitudes, air temperatures are increasing at least two times faster than the global average due to Arctic amplification [2–6]. Arctic change has the potential to accelerate global climate change; one key mechanism is through impacts on carbon (C) cycling within the permafrost region [7–10]. Permafrost (perennially frozen ground) is found within 22–24% of the exposed land surface of the Northern Hemisphere [11–14] and is a significant, climate-sensitive component of the global C cycle. At least 1460–1600 Pg C (1 Pg = 1 billion tons) of soil organic C has accumulated in the permafrost region over the Late Pleistocene and Holocene due to freezing temperatures and waterlogged soils, with another approximately 1000 Pg C likely present in deep terrestrial and subsea deposits [10,13–15]. Record high permafrost temperatures have already been documented across long-term monitoring sites over the last several decades [4,16]. Under high human greenhouse gas emission scenarios, near-surface permafrost is projected to decrease by 90% by 2300, with much of that long-term loss occurring by 2100 [17].

The permafrost region C pool contains three times as much C as the atmosphere [8]. The impact of warming and permafrost loss on global climate change depends on: how much of this C is released to the atmosphere as greenhouse gases while permafrost degrades; the timescale of the release; the proportion of the release of CH_4_ versus CO_2_; and lastly, how much of this release is offset by increased plant biomass and subsequent inputs to the soil C pool [7,10,18–22]. Long-term soil C losses of significant magnitude that can affect climate are expected to arise from C that is cycling on centennial to millennial scales, since this old C comprises the bulk of permafrost soil C. Radiocarbon content of soil organic C can be used to estimate the timescales of C cycling—the amount of time since C was fixed via photosynthesis and stored in various ecosystem pools [23]. Carbon that is actively cycling on annual to decadal timescales will reflect the bomb enrichment of atmospheric radiocarbon in recent decades, whereas C that is cycling on the order of hundreds to thousands of years will have undergone radiocarbon decay and will be depleted in radiocarbon [24,25]. The transfer of old soil C to the atmosphere is expected to occur as a result of warming and permafrost thaw and the microbial decomposition of organic C [7,8]. Importantly, this highly significant change in ecosystem C cycling should be detectable in the radiocarbon values of ecosystem and soil respiration, which can indicate the age of metabolized C. The large range in radiocarbon in permafrost region soils provides a sensitive fingerprint for detecting the loss of old C in ecosystem respiration in response to warming and permafrost thaw.

The radiocarbon content of ecosystem respiration (*ecosystem respiration radiocarbon*) represents a mixture of sources, each with its own radiocarbon value. Ecosystem respiration is derived from autotrophic (plant) respiration and heterotrophic (primarily microbial) decomposition of organic matter. Thus, changes over time in ecosystem respiration reflect atmospheric radiocarbon changes as influenced by the global C cycle and increasing fossil fuel emissions, in combination with changing environmental conditions that shift the contribution of individual plant and soil source pools to overall ecosystem respiration [26]. This idea that ecosystem respiration reflects changes in ecosystem C cycling in response to environmental change has been tested here using a combination of field measurements of isotope ratios, C fluxes and environmental drivers. These were applied in a tundra ecosystem undergoing warming and permafrost thaw, either exposed to experimental soil warming and drying, and/or already undergoing regional environmental warming and destabilization of permafrost [22,27–30]. This unique approach of partitioning ecosystem respiration by incorporating environmental drivers to constrain the contribution of different C sources improves our understanding of drivers of old soil C release to the atmosphere. Previous studies in this system highlighted mechanistic drivers of ecosystem respiration radiocarbon that varied over relatively short periods (2–3 years). Here, we examined the complete time series in order to gain long-term insight into changes in permafrost C dynamics that are unfolding in a warmer world.

## 2. Methods

### (a) Site and measurement locations

The Arctic Carbon and Climate (ACCLIMATE) observatory is characterized by moist acidic tundra within the Eight Mile Lake watershed (63°52′42.1′′ N, 149°15′12′′ W; 670 m.a.s.l.), west of Healy, Alaska [27,31]. Vascular plant cover is dominated by the tussock-forming sedge *Eriophorum vaginatum* and the deciduous and evergreen shrubs *Betula nana*, *Vaccinium uliginosum*, *Rubus chamaemorus, Rhododendron groenlandicum* and *Vaccinium vitis-ideae*. Non-vascular biomass is dominated by mosses and lichens [32–34]. Soils are classified as Gelisols [35], with a thick organic horizon on top of cryoturbated mineral soil, which has accumulated 55–69 kg C m^−3^ [36–38]. The long-term permafrost temperature record on site indicates warming of the permafrost since the mid-1980s, which led to ground subsidence [39–41] and alteration of the surface hydrology, creating a mosaic with different levels of disturbance [27,31,42]. Observations across these differing levels of permafrost thaw that are occurring as part of regional warming are referred to as the **Gradient** site.

Within this context, a warming experiment, established in 2008 and called Carbon in Permafrost Experimental Heating Research (CiPEHR), used a novel approach to warm the deep soil and degrade permafrost: the installation of snow fences to trap snow as it was redistributed around the landscape by winter wind [43]. Increased snow depth maintained elevated soil and permafrost temperatures because the snow acted as an insulating layer between the soil and the extremely cold air. The accumulated snow was shovelled from the snow fences in early spring, so that the water input and the timing of snowmelt of the treatment was similar to the control plots. This design successfully warmed soils and increased growing-season depth of ground thaw by greater than 400% by 2021, degrading an increasing amount of permafrost each year [44]. Permafrost degradation is intimately related to changes in surface hydrology because the loss of ground ice causes ground surface subsidence. Interactions between thaw and soil moisture were examined with a water table manipulation (DryPEHR) established in 2011 within the footprint of the soil warming treatment of CiPEHR [45]. This manipulation altered surface moisture by actively pumping perched water out of plots where barriers had been installed down to the permafrost surface. After 2018, ground subsidence was so great (up to approx. 75 cm in some plots) [46] that water pumping was stopped. At that point moving forward, plots that had randomly been placed at the outset of the experiment and assigned a treatment were now treated as individual measurement plots arrayed across a gradient of thaw depth (TD) and water table, defined by both the experimental treatments and the surface hydrology dictated by the resulting ground subsidence [29,47]. Together, the set of measurement plots at Gradient, CiPEHR and DryPEHR were analysed to develop a mechanistic understanding of the ecosystem sources contributing to C losses following warming and permafrost thaw, and how these dynamics changed over time.

### (b) Ecosystem and soil respiration

*Ecosystem respiration* ^14^CO_2_ and ^13^CO_2_ isotope ratios were measured using a modified dynamic flow chamber system [22,27,29,30]. At each measurement plot, permanent collars (25.4 cm diameter) remained inserted in the soil/moss surface, and dark, 10 l plastic chambers were placed over the collars and air was drawn through a gas analyser. To remove background CO_2_ and to collect ecosystem respired CO_2_ alone, the air stream was scrubbed with soda lime at a rate similar to that of soil CO_2_ efflux until two to three chamber volumes of air had passed through the scrubber, when the CO_2_ remaining in the chamber system was almost exclusively ecosystem respired CO_2_. The air stream was then diverted through a 13× molecular sieve to trap CO_2_ until approximately 0.5–1.0 mg of CO_2_-C was adsorbed. In the laboratory, the molecular sieve traps were heated to 625°C to desorb CO_2_ [48]. Carbon dioxide was then purified, graphitized and analysed for ^14^C and ^13^C. The ^13^C isotope ratios were used in the final analysis to correct for incomplete scrubbing of atmospheric CO_2_ in the chamber-sampler system based on the known difference between atmospheric and ecosystem respiration ^13^C values [24,27,49–51]. Final ^14^C values are expressed as *Δ*^14^C, with the value of 0‰ defined as the ^14^C/^12^C isotope ratio of a wood standard from 1890 [52,53]. Radiocarbon measurements were performed at the Keck Carbon Cycle Accelerator Mass Spectrometer Facility at the University of California, Irvine, and at the Arizona Climate and Ecosystems (ACE) isotope laboratory at Northern Arizona University.

Soil pore space ^14^CO_2_ content (*soil respiration radiocarbon*) was measured from gas samples collected from permanently installed soil gas wells located at a variety of depths within the soil profile (10–50 cm) with a majority of samples taken at 10 and 20 cm depths. The gas wells were made of 1/8′′ ID stainless steel tubing, perforated and covered with mesh at the sampling depth and extending above ground to fittings with gas-tight stopcocks. Air was pumped from each gas well at 0.5 l min^−1^ for about a minute through a 13X molecular sieve to quantitatively trap CO_2_, with the same laboratory procedures used to purify and analyze for ^14^C. In the case of soil gas, no ^13^C isotope correction was used, unlike what was done for above-ground ecosystem respiration, because intrusion of atmospheric air was determined to not be an issue for soil profile gas.

### (c) Annual plants and soil organic matter

Annual plants were sampled and analysed for radiocarbon as a time-integrated snapshot of the changing atmospheric radiocarbon ratio [54–57]. Away from urban areas, the background level of atmospheric radiocarbon was above pre-industrial levels for our study period due to the production of radiocarbon in the atmosphere from past nuclear weapons testing. Atmospheric radiocarbon values have been dropping since the early 1960s when above-ground testing stopped due to the exchange of C with the oceans and the biosphere, and the continued dilution of atmospheric radiocarbon by fossil fuel emissions. Annual plants integrate this signal because photosynthesis provides a cumulative record of the radiocarbon of the local atmosphere integrated over the many-months growing period of the plant. Each year, we collected the annual plant *Matricaria discoidea* at locations surrounding the study site and away from potential local fossil fuel sources, and analysed the above-ground tissue for radiocarbon. Early in our sampling record (2003–2006), we made instantaneous measurements of atmospheric radiocarbon at the study sites using molecular sieves or glass flasks to trap CO_2_ from air at 2 m height before switching to annual plants in 2006 for the rest of the time series. Solid samples (plants) were combusted in sealed quartz tubes with cupric oxide to produce CO_2_, which was then purified and analysed as described previously. We also compared the measurements from our local site with a clean-air atmospheric CO_2_ flask record collected at Utqiagvik, Alaska, about 850 km away [58].

Soil organic matter was sampled and analysed for radiocarbon as part of several previous studies at the site that focused on the accumulation of soil/permafrost C over time. These data are shown here to illustrate the source of old carbon to ecosystem and soil respiration radiocarbon [36]. Methods are described in detail elsewhere but, briefly, organic carbon was collected at different depths in the soil profile at control locations at the site. Plant macrofossils or bulk soil organic carbon samples were combusted in the laboratory as described for annual plants and analysed for radiocarbon.

### (d) Environmental measurements

Environmental drivers of ecosystem respiration radiocarbon were analysed at CiPEHR only because environmental data at that site were paired directly with the permanent collars used for radiocarbon sampling. Detailed descriptions of C flux and environmental measurements are available for the CiPEHR warming experiment [43,45,59]. Out of a larger suite of site environmental variables, a subset was selected as drivers to predict ecosystem respiration radiocarbon values based on a previous analysis of autochamber ecosystem CO_2_ fluxes [60]. In brief, soil temperature (°C) was measured at 5, 10, 20 and 40 cm every half hour using type T copper-constantan thermocouples and recorded on a data logger (CR1000; Campbell Scientific), and soil volumetric water content (VWC; %) was recorded every half hour in each plot with TDR probes (CS 616; Campbell Scientific) integrated from 0 to 20 cm soil depth. Readings from most sensors were averaged over 30-min intervals. Precipitation was measured with a HOBO Onset station during the growing season (GS, Bourne, MA, USA). TD was measured weekly as the distance (in centimetres) from the moss/surface layer to the frozen layer within the soil. Water table depth (WTD) was measured as the distance (in centimetres) from moss/soil surface to the water table surface perched on the frozen soil within nine 3-inch diameter PVC wells. Normalized difference vegetation index (NDVI) was used to measure plant biomass at the plot scale using a hand-held camera (Tetracam, Chatsworth, CA, USA), and independently a point-intercept method was used to estimate plant biomass [61]. Ground subsidence as a result of warming and the loss of ground ice was measured with differential GPS [46]. Net ecosystem C exchange (NEE) is the balance between two major opposing processes: CO_2_ uptake by primary producers (gross primary production, GPP), and CO_2_ losses by ecosystem respiration, comprising autotrophic plant respiration and heterotrophic respiration [62]. NEE was measured using clear autochambers that sequentially measured CO_2_ fluxes from each plot [63,64]. NEE measurements were separated into GPP and ecosystem respiration based on standard gap-filling techniques using environmental scalars: light and temperature.

### (e) Statistical analysis

The slopes of atmospheric, ecosystem respiration and soil respiration Δ^14^C over the 19-year time series were calculated with mixed linear regression models using the package nlme in R [65,66]. We limited our analyses to data collected during the GS at this site (May through September) to avoid low *Δ*^14^C values that can occur when the plants are dormant and surface soil is frozen [30,67,68]. Overall, we had 46 observations of atmospheric CO_2_, 867 observations of ecosystem respiration and 472 observations of soil respiration CO_2_. Given the differences in sample size and variance, models of each Δ^14^C variable were run separately. For ecosystem and soil respiration, a random intercept of sample ID was included to take into account the repeated measurements from individual collars. A random slope of sample ID was also tested, but did not improve the model AIC and so was not included in the final model. The variance of ecosystem and soil respiration Δ^14^C increased over time, so a variance structure was included in the models that had the variance increase exponentially with the year of sampling (varExp). Additionally, the inclusion of a temporal autocorrelation structure was tested and retained if it decreased the model AIC by more than five units. For the soil respiration dataset, a ‘corAR1' temporal autocorrelation structure was included. Two models were run for ecosystem respiration: one with only Year as a predictor to investigate overall trends, and one with Year, Location, and a Year by Location interaction to investigate whether the time trend from the gradient site differed from those of the two soil warming experiments: CiPEHR and DryPEHR. With fewer soil respiration data and shorter time series at any given site, we did not model a Year by Location interaction, which had the effect of grouping all sites together. Instead, two models were run for soil respiration: one with only Year as a predictor and another with Year, Depth, and a Year and Depth interaction. We tested whether the modelled slopes of ecosystem and soil respiration over time were significantly lower (more negative) than the slope of the atmosphere over time using a one-tailed *Z*-test (*α* = 0.05) based on slopes and standard errors estimated from the linear mixed models described above [69]. The ecosystem and soil respiration datasets included a number of very negative (old) radiocarbon values, especially towards the end of the time series. To investigate the extent to which the estimated slopes were driven by these negative radiocarbon values, all of the statistics described above were also performed without these values by including only the data points within one standard deviation of the median as a sensitivity test.

The environmental and C flux measurements described previously for CiPEHR were used as input variables for Gradient Boosted Regression Tree statistical models with ecosystem respiration radiocarbon as the response variable. The models were run in R with the package gbm [70]. A model was fit for the entire dataset using the following variables that were aggregated for the week preceding the radiocarbon measurement, unless another time aggregation is specified: ecosystem respiration, GPP, full GS GPP, soil temperature (means and standard deviations as separate variables for 5, 10, 20, 40 cm depths), air temperature (mean and standard deviation at 2 m height), VWC (mean and standard deviation), WTD, TD, NDVI (mean), GS WTD (aggregated from May 1 to 2 weeks prior to radiocarbon measurement), precipitation (aggregated at one week, two weeks, and from May 1 to two weeks prior to radiocarbon measurement), subsidence, and plant biomass derived from point-intercept measurements. As detailed earlier, this subset of variables was selected based on extensive prior analysis of autochamber CO_2_ fluxes and strikes a balance between the total number of variables used within the regression tree model and the number of radiocarbon observations. For the model, 75% of the data were used for model training and the remaining 25% were used for validation. Optimal parameters (number of trees, interaction depth, shrinkage and minimum observations per node) were determined by comparing model performance over a grid of possible parameters. To reduce the likelihood of overfitting, the model was re-run with a subset of the best predictor variables, which accounted for 85% of the model relative influence. Model fits were evaluated using linear regression between the observed and predicted radiocarbon isotope ratios. The radiocarbon dataset was not normally distributed and included a number of very low, negative values. We ran a separate regression tree model with the same variables and procedures as described above but included only radiocarbon respiration values within one standard deviation of the median, which had the effect of removing the very negative values. This sensitivity analysis was done to test whether these extremely negative radiocarbon values were behaving differently in response to changing environmental variables when compared with observations closer to the median. There were not enough negative excursions to analyse those data alone using the regression tree approach.

## 3. Results

### (a) Atmosphere

**Figure 1.**
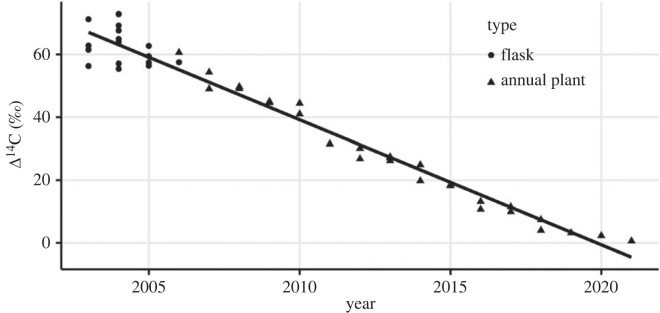
Atmospheric measurements of radiocarbon in interior Alaska measured instantaneously (flasks) or integrated over the summer GS (annual plant). Positive values indicate bomb radiocarbon.

Atmospheric radiocarbon ratios declined over the 19-year measurement period, with initial values at or above 65‰ in 2003 and declining below 0‰ after 2020 (figure 1). Instantaneous air measurements tended to be somewhat more variable than the annual plant samples, which integrated atmospheric radiocarbon over the course of the GS. The annual −4‰ decline in atmospheric radiocarbon ratio observed here (table 1) mirrors patterns observed regionally [58] and globally [71].

**Table 1.** The slopes ± s.e. from linear regression models with Year as a predictor for the ^14^C of the atmosphere, ecosystem respiration and soil respiration datasets. ‘All' refers to the slope when data were pooled across locations, while the site name refers to results from regressions that included a Year by Location interaction (for ecosystem respiration), or a Year by Depth interaction (for soil respiration). *p*-values reflect results of one-tailed *z*-tests (*α *= 0.05) to determine whether slopes were significantly lower than the observed atmospheric decline. Statistics that were significant (less than *p* = 0.05) were shown in bold.

dependent variable	location	slope	*p*-value (*z*-test)
atmospheric Δ^14^C		−4.0 ± 0.1	
ecosystem respiration Δ^14^C	all	−6.3 ± 0.2	**<0**.**0001**
	gradient	−5.7 ± 0.9	**0**.**02**
	CiPEHR	−4.8 ± 0.8	0.15
	DryPEHR	−12.0 ± 1.7	**<0**.**0001**
soil respiration Δ^14^C	all	−6.5 ± 0.8	**0**.**0008**
	10 cm	−5.4 ± 1.0	0.07
	20 cm	−7.7 ± 1.3	**0**.**002**

### (b) Ecosystem and soil respiration

**Figure 2.**
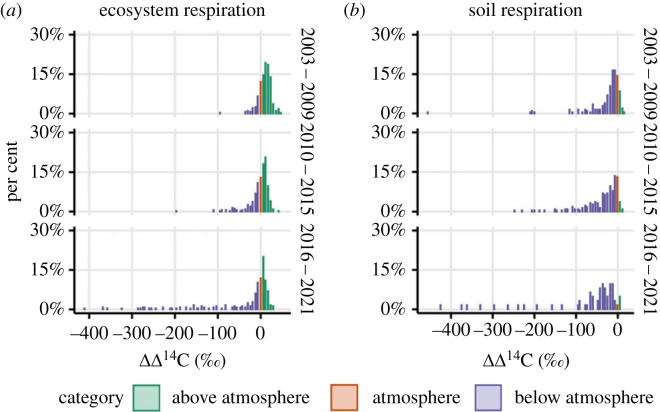
Histograms showing distribution of ecosystem respiration (*a*) and soil respiration (*b*) radiocarbon values through time, grouped into 6-year intervals. All ecosystem and soil respiration observations shown here have been detrended against the atmospheric slope through time resulting in atmospheric values of each year centred on 0. The categories represent radiocarbon values that were either: greater than 3‰ above the atmosphere in the year of measurement (green), within 3‰ of the atmosphere (red) or greater than 3‰ below the atmosphere (blue). These cut-offs represent the instrument precision required to distinguish an ecosystem or soil respiration radiocarbon value as different from the atmosphere in the year of measurement. Each bar represents the proportion of observations in a given year group binned into 6‰ intervals.

Across the full dataset of ecosystem respiration, 56.3% of the observations (*n* = 867) had values above the atmospheric value (greater than 3‰ above) in the year they were measured, 12.3% had values at the atmospheric value (within 3‰) and 31.4% had values below the atmospheric value (greater than 3‰ below). These bins were defined by instrument precision of approximately 3‰, and thus separate values that are indistinguishable from the atmosphere versus those that differ from the atmosphere. Mixture values above the current-year atmospheric radiocarbon value illustrate a major contribution from the decomposition of materials with additional legacy bomb radiocarbon—materials that have resided in the ecosystem for years to decades. Fast-cycling C, such as the majority of plant and rhizosphere respiration, has a radiocarbon value close to, or at, the current-year atmospheric value as photosynthesis is effectively labelled by the current year [22]. Slow-cycling C, such as organic matter preserved in soil, is a source of old C that when released contributes respiration with radiocarbon values below the current atmosphere. If enough time has passed for radioactive decay, respiration of this C can have negative radiocarbon values. All observations of ecosystem and soil respiration contain a mixture of these sources, representing a range of past atmospheric values combined with radioactive decay, depending on the residence time of the mixture of C released back to the atmosphere [53]. The proportion of ecosystem respiration measurements with radiocarbon values below the atmosphere in the year they were measured changed over time: 14% of measurements were below the atmosphere prior to 2009, 34% were below between 2010 and 2015, while 47% were below after 2015 (figure 2).

For the dataset of soil respiration measured within the soil profile (*n* = 472), there were lower radiocarbon values (mean = 0‰ ± 3) as compared with ecosystem respiration (mean = 21‰ ± 2). This difference was because heterotrophic soil respiration sources were proportionally greater relative to plant respiration sources within the observed mixture, as soil respiration was not influenced by above-ground plant respiration or the decomposition of surface plant litter. For soil respiration, 7.2% of the observations had values above the atmospheric value (greater than 3‰ above) in the year they were measured, 11.7% had values at the atmospheric value (within 3‰) and 81.1% had values below the atmospheric value (greater than 3‰ below).

Both the ecosystem and soil respiration datasets featured observations where old C respiration was a dominant contributor. Nine per cent of the ecosystem respiration dataset and 23% of the soil respiration dataset had mixture values more than 50‰ lower than the atmospheric value (approx. negative radiocarbon values) in the year they were measured. Even lower respiration observations of −200‰ to −400‰, corresponding to radiocarbon ages of several thousand years old, were observed after 2015. These negative values represent the contribution of sources significantly older than that mixed with younger sources. Negative values were observed both within the soil profile (soil respiration) and also emitted at the ecosystem–atmosphere interface (ecosystem respiration).

**Figure 3.**
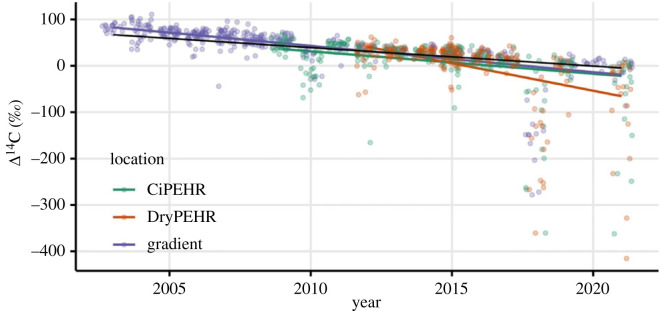
Time series of ecosystem respiration radiocarbon relative to changes in atmospheric radiocarbon. The black line depicts the slope of atmospheric radiocarbon measured at the ACCLIMATE observatory. The coloured points represent ecosystem respiration measurements from three sites; lines represent their respective slopes based on a linear mixed model. The data points have been jittered on the *x*-axis to increase visibility.

**Figure 4.**
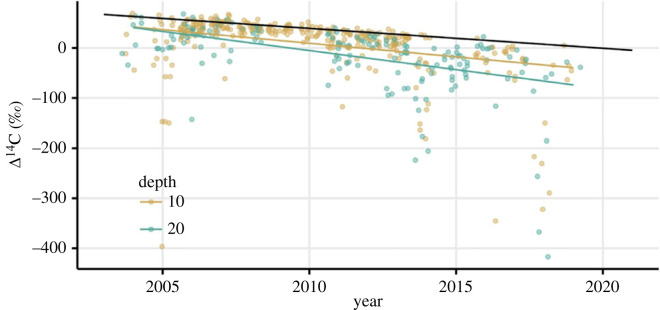
Time series of soil respiration radiocarbon relative to changes in atmospheric radiocarbon. The black line depicts the slope of atmospheric radiocarbon measured at the ACCLIMATE observatory. The coloured points represent soil respiration at 10 and 20 cm depths; lines represent their respective slopes based on a linear mixed model. The data points have been jittered on the *x*-axis to increase visibility.

The 19-year record of ecosystem respiration radiocarbon from ACCLIMATE showed an overall decline over time (figure 3). The decline in ecosystem respiration radiocarbon (−6.3‰ per year, table 1, *p* < 0.0001) across all sites was significantly greater than the −4.0‰ atmospheric decline. While the decline in the warming experiment (CiPEHR) alone (−4.8‰ per year) was not significantly different from the atmosphere (*p* = 0.15), the decline in the Gradient was (−5.7‰ per year, *p* = 0.02), with the drying manipulation (DryPEHR) showing the greatest decline of the three sites (−11.9‰ per year, *p* < 0.0001). The time series of soil respiration radiocarbon from the site shows a similar overall decline (figure 4) that also decreased at a significantly faster rate (−6.5‰, table 1, *p* = 0.008) than the atmosphere. The decline of soil respiration at 10 cm depth (−5.4‰ per year *p* = 0.07) was only marginally significantly greater than the atmospheric decline, while the decline of soil respiration at 20 cm depth was significantly greater (−7.7‰ per year, *p* = 0.002). While the declines in ecosystem and soil respiration have similar slopes, soil respiration radiocarbon values were overall lower than ecosystem respiration values, reflecting a greater contribution from older heterotrophic sources.

Both the ecosystem and soil respiration time series featured a significant number of very low radiocarbon values, and these negative excursions increased across the time series (figure 2). To determine the influence of these negative excursions on the overall time series trends, a sensitivity test was performed using a similar regression analysis with time, but with radiocarbon values >1 s.d. lower than median removed. For ecosystem respiration, the negative slope with time was still significantly lower than the atmospheric decline when all sites were combined (−4.3‰ per year, *p* = 0.01), but the statistical significance was weaker when sites were analysed individually. For soil respiration, the negative slope with time (−4.1‰ per year) was not statistically different from the atmospheric decline when all sites were combined, and for 10 cm (−4.1‰ per year) and 20 cm depths (−3.7‰ per year), individually. The negative excursions are a real feature of the time series both for ecosystem and soil respiration; this sensitivity test suggests that those excursions contribute to the radiocarbon trends over time, but are not the only drivers.

### (c) Soil organic carbon

**Figure 5.**
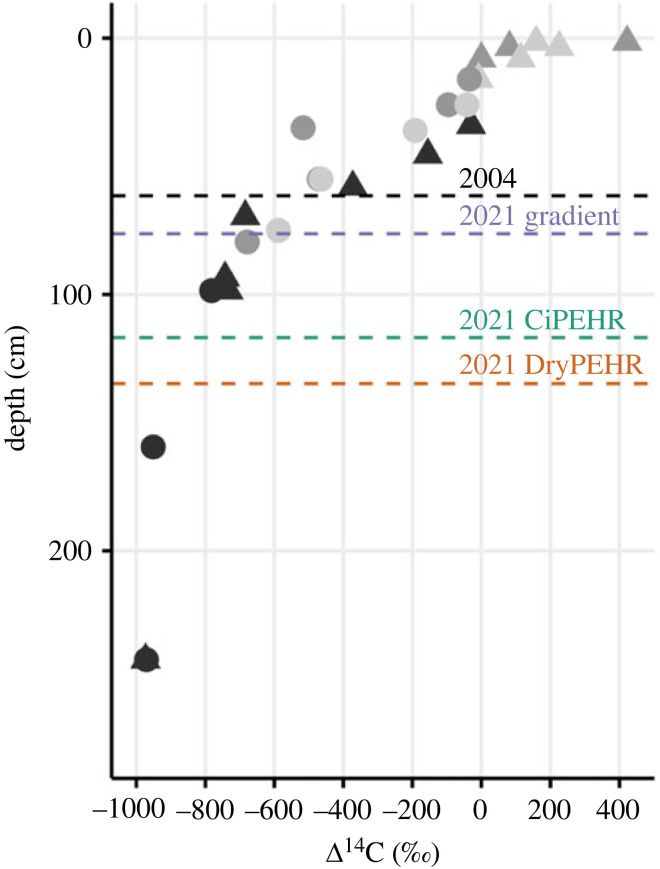
Radiocarbon values of soil organic matter with depth in the soil/permafrost profile. Circles denote bulk soil measurements, and triangles denote measurements from isolated plant macrofossils. Light and medium grey data points are from Hicks Pries *et al*. [36] and darker grey data points are from Hutchings *et al*. [37]. Dashed lines represent the average end-of-season TD (active layer) in 2004 and 2021 coloured by location.

Soil C accumulation has been previously studied at the ACCLIMATE site using C content and radiocarbon age of organic matter to determine rates of accumulation [36,37]. Owing to the upward accumulation of surface organic C along with aeolian deposition of mineral soil, the radiocarbon content of organic C gradually decreases with depth (figure 5). Elevated radiocarbon (positive values) in surface organic C reflects enrichment from bomb radiocarbon, with observed values above +200‰ reflecting photosynthesis and soil organic C accumulation over the past several decades. Negative radiocarbon values deeper in the soil profile ranging from −400‰ to −950‰ below 1.5 m depth represent significant radioactive decay. Macrofossil radiocarbon values around 1 m depth correspond to calendar ages greater than 10 ka BP, and organic C has accumulated over many metres of soil at this site for greater than 40 ka [37].

### (d) Environmental drivers

**Figure 6.**
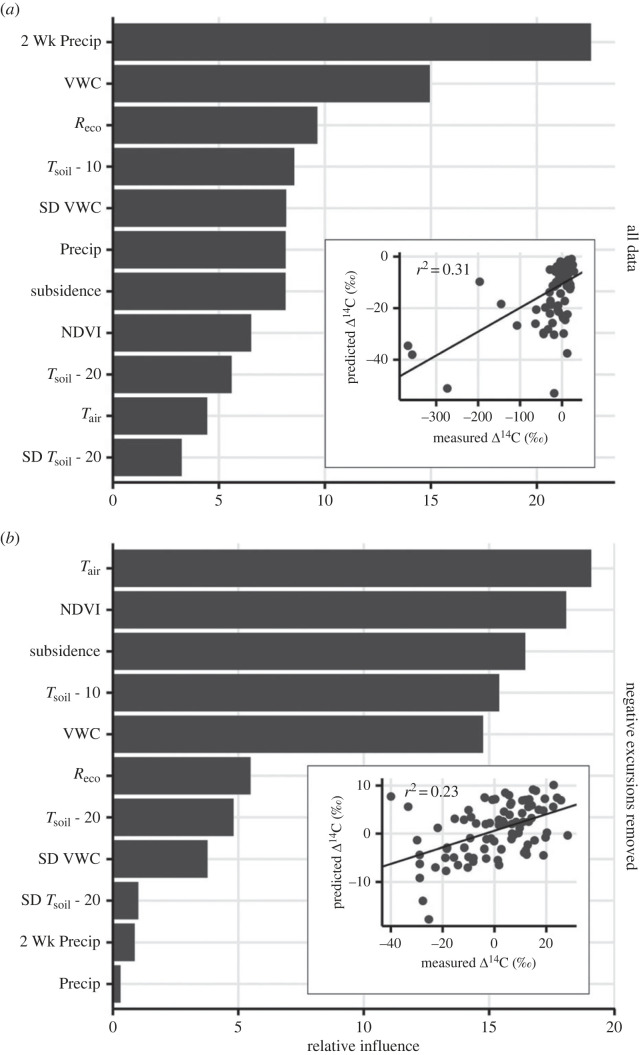
Results of a gradient boosted regression tree analysis predicting ecosystem respiration radiocarbon measurements from the CiPEHR warming experiment using paired environmental variables. The relative influence values show the frequency with which a particular environmental variable occurred in the tree analysis. The inset to each panel shows the modelled versus the observed radiocarbon value using the best-fit boosted regression tree model. (*a*) shows the results of the analysis using all available data, while (*b*) shows the results of a sensitivity analysis after negative radiocarbon excursions were removed.

To examine drivers of ecosystem respiration radiocarbon values, we performed a boosted regression tree analysis on the soil warming experiment plots for which paired ecosystem respiration radiocarbon data and environmental measurements were available. When all radiocarbon values were included, the boosted regression tree analysis identified: precipitation (two-week aggregation) and VWC as the top two environmental drivers, with relative influence scores greater than 15 (figure 6*a*). Ecosystem respiration and soil temperature (10 cm) were identified as the next two most influential drivers. A regression between modelled and observed ecosystem respiration radiocarbon (figure 6*a*, inset) showed that the model explained about 30% of the variation, but that the negative radiocarbon excursions were not predicted well by the dynamics of the environmental drivers. Furthermore, the structure of the observed data did not lead to a random distribution of residuals around the model-observation regression line. To address this, a second boosted regression tree analysis was performed on a dataset that excluded the negative radiocarbon excursions (greater than 1 s.d. lower than median). This analysis identified air temperature and NDVI as the top two influential drivers (figure 6*b*), with ground subsidence, soil temperature (10 cm) and VWC following sequentially thereafter as influential variables, all near or above relative influence scores of 15. The regression between modelled and observed values in this restricted case led to a random distribution of residuals (figure 6*b*, inset) even though the shortened range of radiocarbon values overall caused a slight decrease in the model goodness-of-fit (*r*^2^ = 0.23).

## 4. Discussion

This one-of-a-kind, 19-year time series of radiocarbon from a changing permafrost ecosystem revealed a decline in atmospheric radiocarbon value over time. The decline was significantly greater in the time series of radiocarbon values of soil and ecosystem respiration, the C flux from the ecosystem back to the atmosphere. The current annual decline in atmospheric radiocarbon that was observed at the site is primarily due to fossil fuel combustion, whose radiocarbon-free emissions dilute the global atmospheric radiocarbon ratio [71]. Because the atmosphere is a relatively well-mixed C pool [72], the −4‰ per year decline at our site matched exactly with regional and global records [58,71]. Aside from the main effect of fossil fuels on atmospheric decline, ecosystem respiration has been returning bomb radiocarbon back into the atmosphere over the past 50 years. Ecosystem respiration radiocarbon ratios have been higher than atmospheric radiocarbon ratios for several decades. The disequilibrium between ecosystem respiration and the atmosphere is decreasing, starting from soon after the 1960s bomb peak and moving towards the present day [71,73,74]. The atmospheric decline of −4‰ per year should maintain or even become more negative if fossil fuel use grows along with less bomb radiocarbon returning from ecosystems in the future.

Ecosystem respiration represents a mixture of sources, and thus its radiocarbon value is not a fixed calendar date [25], but instead an approximation of the C turnover times of the various sources [24,26]. Mixture values above the current-year atmospheric radiocarbon value illustrate a major contribution from bomb radiocarbon derived from C within the ecosystems (figure 5) that was fixed over the past 60 years [75–77]. Mixture values below the atmosphere in the year of sampling reflect older C, some which is old enough to reflect radioactive decay (figure 5). Respiration values similar to the atmosphere in the year of sampling likely reflects the fastest turnover C that was fixed the same, or the previous year, although it is not possible to rule out a mixture of older and younger C that matches the atmosphere using the radiocarbon measurement alone [78].

Steady-state ecosystem C dynamics serve as a null hypothesis against which we can evaluate the ecosystem and soil respiration radiocarbon time series from our changing permafrost ecosystem [24,79]. In steady state, ecosystem C storage is constant throughout time with ecosystem respiration outputs balanced by plant production inputs. The atmospheric decline of −4‰ per year (figure 1) represents the theoretical upper limit of decline that should be observed for ecosystem respiration over time in a steady-state ecosystem. And in fact, the decline of ecosystem respiration through time should have a somewhat less negative slope, since autotrophic respiration and fast-cycling C labelled by the current-year atmosphere could be 50–75% (but not 100%) of ecosystem respiration returned back to the atmosphere [22,29,50,80]. The remainder of the return C flux (25–50%) is derived from ecosystem pools with longer C turnover times [22]. Slow turnover C pools have slower year-to-year changes in radiocarbon values since new atmospheric C inputs are buffered by older C already contained in the pool [73,79]. If the atmosphere returned to pre-bomb levels under steady-state conditions, ecosystem respiration radiocarbon would eventually converge back closer to the atmospheric value given enough time. In the actual world, where the atmospheric radiocarbon ratio is being continuously diluted by fossil fuel emissions that is unlikely to happen anytime this century. The future disequilibrium offset (ecosystem respiration radiocarbon *higher* than the same-year atmosphere) will be dependent on the magnitude of human C emissions with a greater disequilibrium arising under higher fossil fuel use (e.g. RCP8.5/SSP5-8.5) [71,81].

We hypothesized that the degradation of permafrost in a warming Arctic would increase the contribution from old soil C sources to total soil and ecosystem respiration, and be reflected in declining radiocarbon values [31,82,83]. Such a fingerprint of warming and permafrost C destabilization (non-steady-state conditions) would manifest as respiration radiocarbon values below atmospheric values, with the potential for these observations to increase in frequency as warming and permafrost thaw progressed (figure 5) with more old carbon exposed to decomposition. This extended dataset clearly demonstrated the presence of old C release throughout the time series, with the frequency of unequivocally old C (mixtures below atmospheric radiocarbon values) increasing from the beginning of the time series to present (figure 2). Respiration of old C pools (figure 5) not only influenced average soil and ecosystem radiocarbon values, but also manifested as sporadic pulses where old C was the dominant contributor to the overall respiration mixture, resulting in very negative radiocarbon values. Old C is also contributing somewhat to mixtures whose values fall above the atmosphere in the year of sampling, but disentangling the proportional contribution requires other ecosystem measurements in combination with radiocarbon.

Analysed together, the time series showed a greater yearly decline in radiocarbon values of ecosystem and soil respiration across the dataset, relative to atmospheric −4‰ per year. This was largely true whether the analysis was performed with the entire dataset, or with the negative radiocarbon excursions removed. The presence of substantially old C (negative radiocarbon values or mixtures below the atmosphere in the year of sampling) in any single instantaneous measurement of ecosystem and soil respiration in and of itself is not necessarily diagnostic of the destabilization of permafrost C pools given short-term fluctuations in environmental and soil conditions. This is because even with steady-state dynamics, some fraction of the respiration flux from ecosystems back to the atmosphere should contain old C, albeit at a very small proportion relative to the slow turnover of the oldest C pools [24]. Nonetheless, even a small amount of very old C can greatly decrease average radiocarbon values of a mixture [79]. However, the observed decline in ecosystem and soil respiration over the entire time series that was statistically greater (more negative) than the decline in the atmosphere is a fingerprint of an increasing contribution from old C to ecosystem respiration, beyond what could be supported with steady-state conditions.

The greater decline in ecosystem and soil respiration when compared with the atmospheric decline was a feature of the entire dataset, and in some cases the response of different sites could be distinguished. The ecosystem respiration radiocarbon decline was significantly greater than the atmospheric decline for all sites combined, the gradient and drying experiment by themselves, but not for the warming experiment by itself (table 1). This is partly due to the nature of the old C release as pulse events that occurred sporadically across sites, as well as the shorter time series (fewer number of years) for individual sites (warming, drying) as compared with the entire dataset. The shorter time series reduces the statistical ability to detect differences in slope (figure 3). The increase in observation variance through time in the dataset actually decreased the statistical power to detect differences in slope. Accounting for this widening variance structure in the statistical analysis was required in order to obtain normal residual distributions. Despite these challenges to the statistical approach, most slopes were significantly greater than the atmospheric decline, strongly indicating that old permafrost C pools are destabilizing and releasing more C to the atmosphere, moving away from conditions that supported those pools over the Holocene [36].

Of course, the decrease in radiocarbon value of the ecosystem and soil respiration mixtures alone does not unequivocally distinguish between an increase in the contribution of old C sources versus the declining contribution of either plant respiration or decomposition of C pools enriched in bomb radiocarbon [27,84]. However, other data from the site including previous isotope partitioning studies point towards increasing contributions, not decreasing, from plant C sources [28,85]. Long-term eddy covariance measurements of ecosystem C fluxes at the site show monotonic increases in plant carbon uptake (GPP) and increasing ecosystem respiration through time from 2004 to present [41], with the ecosystem overall losing net C to the atmosphere. Isotope partitioning studies using Bayesian approaches to analyse short (2–3 year) sections of this time series also confirmed an increase in the contribution of absolute fluxes from old C pools with both natural thaw and experimental warming [28–30]. Together, these observations show that the C pools contributing to the ecosystem respiration radiocarbon mixtures cannot be in steady-state with respect to the turnover times that stored the C in the first place. Furthermore, the extremely negative excursions away from steady-state values occur with increased contribution of old C (more negative ecosystem respiration mixture). These excursions occur in pulses throughout the time series and these pulses are increasingly common in the latter half of the time period (figure 2). At the same time, the sensitivity analysis that excluded the negative radiocarbon excursions still showed the greater decline over time when compared with the atmosphere, albeit the pattern was weaker than for the full dataset. This suggests there was also a continuous increase in the baseline of old C contribution; the pulses were not the only time when increasing old C loss was contributing to soil and respiration fluxes. At the same time, this illustrates the importance of the negative excursions to the overall observed pattern. Together, these observations are consistent with the hypothesis that older C pools contributed more to the observed mixture over time as a fingerprint of warming and permafrost soil C loss.

Boosted regression tree analysis of environmental controls over ecosystem respiration radiocarbon from the warming experiment pointed towards indices of moisture (e.g. precipitation, VWC, VWC SD), soil temperature (10 cm) and ecosystem respiration flux rates as the driving variables with the largest relative influence (figure 6, top). Increases in the contribution of old C to the observed mixtures were expected with increased soil temperature and permafrost thaw (figure 5) [27]. The additional influence of moisture suggests that old C can still be environmentally protected by anoxic conditions within the water table perched on the permafrost surface, which saturates the lower soil profile at many measurement locations. The influence of the water table in restricting old C loss was demonstrated previously in isotope partitioning studies from these same sites [29] and other peatland sites [86], and was also supported by the regression tree sensitivity analysis (figure 6, bottom). When the negative excursions were filtered from the radiocarbon dataset, moisture variables were replaced by air temperature, plant biomass (NDVI), ground subsidence and soil temperature (10 cm) as the top four variables with highest relative influence, while moisture (VWC) declined in importance as the variable with the fifth highest relative influence. The variables that controlled the restricted dataset were related to soil temperature/thaw and to variables that controlled the contribution of plant respiration to the overall respiration mixture (e.g. air temperature, NDVI) [22,28]. Plant respiration derived from recently fixed CO_2_ exerts important control over radiocarbon values of ecosystem respiration [28,30], but could not overwhelm the highly negative radiocarbon excursions when the subsurface soil C was drier [45]. Taken together, retaining the negative radiocarbon excursions constrained the model fit better for the negative values at the expense of the model fit for the larger number of observations near the median. This supports a conclusion that there are multiple drivers of ecosystem respiration radiocarbon across the observed range of values with the majority of our time series data driven by TD, soil temperature and NDVI (plant productivity), while the negative excursions being best explained by changing moisture dynamics [87].

The interacting controls of warming and permafrost thaw in combination with soil drying acting to increase old C loss was supported by the previous isotope partitioning study [29] as well as the observation that the radiocarbon time series decline was greatest (most negative slope) for the drying experiment as compared with the warming experiment and the thaw gradient (table 1). This latter evidence is somewhat tempered by the fact that the drying experiment time series was the shortest of all three sites, which can influence slope estimates, and that the drying manipulation was stopped before the end of the time series. Nevertheless, cold temperatures/freezing and soil saturation together acting to slow old C release are protection mechanisms that could shift with warming and permafrost thaw. Widespread warming and permafrost thaw is generally accepted for future climate scenarios as the world warms[17,88–90], but future soil moisture/waterlogging projections are still divergent [91,92]. Heterogeneous surface subsidence where ground ice is lost can lead to both wetter and drier soils at the plot level within the same site undergoing environmental change [46,47], at least during the initial phases of permafrost degradation. Soil saturation is controlled to a large degree by a shallow water table that sits perched on the thaw surface, and which gradually moves downwards in the soil profile following the thaw front through the summer GS. Permafrost thaw and ground ice loss collapses the surface soil into the water table, leading initially to wetter soil conditions. With additional thaw, however, there is the potential for the perched water table to follow the thaw front deeper into the soil profile leaving surface soils drier than they once were. This heterogeneity at the site level is also observed at the landscape level with permafrost thaw causing increases in wetland and lake area, while elsewhere causing aquatic ecosystems to drain [93]. These changes in surface water/wetness also play out against a backdrop of an intensifying hydrologic cycle in a warmer Arctic [94] making projections of future trajectories of the soil waterlogging protection mechanism a challenge.

## 5. Conclusion

The time series of respiration radiocarbon in combination with C flux measurements from this tundra ecosystem unequivocally demonstrates that threshold changes in ecosystem C dynamics are occurring. Because of the size of the C pool, tipping points in permafrost C ecosystems have implications for the future trajectory of climate change [95]. The potential for multiple plausible environmental trajectories in a warmer Arctic is a compelling backdrop for expanding long-term time series of ecosystem and soil respiration radiocarbon in order to detect old C loss. This time series showed that, even with the sporadic nature of old C releases, overall trends through time could reveal the destabilization of permafrost soil C. The presence of the negative radiocarbon excursions also suggests that longer time series will be extremely helpful in order to statistically detect trends within the reality of non-normally distributed observational datasets. This signal of increasing old C release was modified by plant respiration that effectively dilutes the old C signal in individual plots with higher plant biomass, or at times with greater plant productivity and respiration. Thus, it is critical to combine ecosystem and soil respiration radiocarbon observations with C flux measurements either at the plot or site level in order to disentangle changing C source contributions to the overall radiocarbon respiration value mixture. Conversely, radiocarbon respiration measurements can inform ongoing ecosystem C balance measurements at eddy covariance measurement sites. Year-to-year fluctuations in net ecosystem production measured by flux towers alone cannot distinguish between shifts in faster turnover or slower turnover C pools, but changes in these pools have widely different implications for overall long-term ecosystem C storage. Quantifying change in slow turnover C as ecosystems everywhere respond to global environmental change is critical for understanding ecosystem feedbacks to climate change, and radiocarbon remains a unique tool with which to do so.

## Data Availability

Data have been submitted to the Bonanza Creek LTER data portal. At present they have not been issued a DOI, but this should be available shortly. Data are findable at the BNZ LTER Data catalogue by searching for PI = Schuur in advance of the DOI.
